# B-cell subset heterogeneity in the liver during chronic hepatitis B

**DOI:** 10.1007/s12072-025-10930-3

**Published:** 2025-10-03

**Authors:** Abdus Salam, Ulrich Kalinke, Matthias Bruhn

**Affiliations:** 1 Institute for Experimental Infection Research, TWINCORE, Centre for Experimental and Clinical Infection Research, a joint venture between the Helmholtz Centre for Infection Research and the Hannover Medical School, 30625 Hannover, Germany; 2 Cluster of Excellence RESIST (EXC 2155), Hannover Medical School, 30625 Hannover, Germany

Dear Editor,

With great interest, we read the recent study by Li *et al.* on single-cell transcriptomic profiling of B cells in patients with chronic hepatitis B (CHB) [1]. This study analyzed previously published data [2] based on isolated PBMCs and CD45^+^ immune cells from liver biopsies of individuals in different clinical phases of CHB, immune tolerant (IT), immune activation (IA), chronic resolved (CR), acute resolved (AR), and healthy controls (HC). Amongst liver-resident B cells, Li *et al.* identified five B-cell subsets. Activated *FCRL1*^+^ B cells were increased in IA, and exhausted atypical B cells were increased in IT. Atypical B cells are well-described during chronic infections such as hepatitis C [3] and participate in COVID-19 vaccine responses [4]; hence, their presence in the liver caught our attention.

Whereas in the study by Li *et al.*, the authors applied data harmonization to analyze B cells from liver and blood in one approach, we focused selectively on the analysis of liver-resident B cells (Fig. 1a). We noticed that B cells from liver biopsies exhibited considerable heterogeneity, as highlighted by the presence of pre-activated B cells (Fig. 1b), which is a subset that Li *et al.* did not detect in their analysis, likely due to the data harmonization and setting of different clustering parameters. Annotation of these B cells by integrating marker gene expression with immunoglobulin isotype information resulted in the identification of seven subsets, naïve (NBCs), pre-activated 1 and 2, activated (expressing *NR4A1*, *CD69*, *FOSB*, and *CD83)*, germinal center (GC), atypical, and memory B cells (MBCs).

**Fig. 1:**
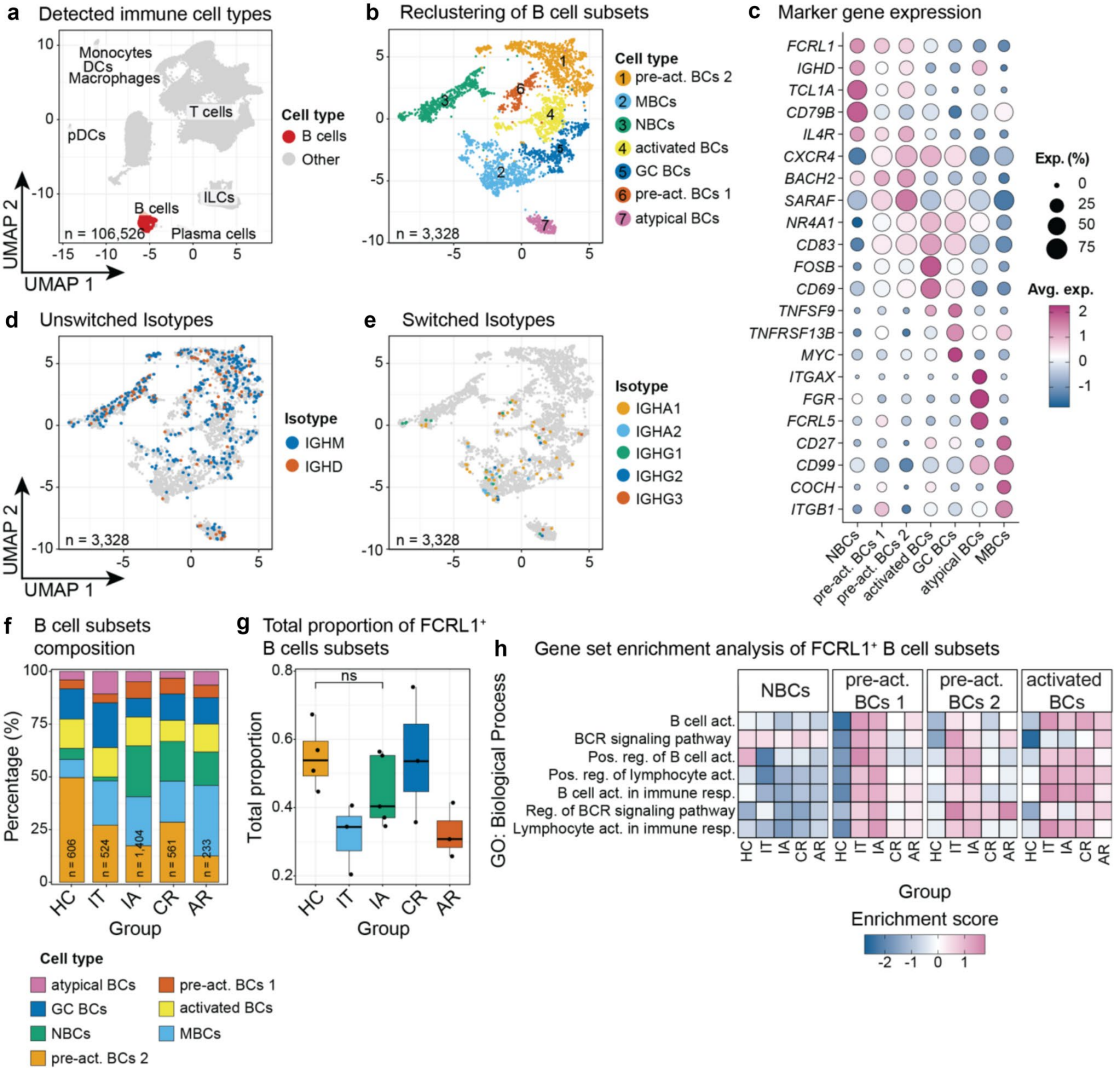
Heterogeneity of liver-resident B cells in different phases of chronic hepatitis B. Bioinformatics analysis of B-cell subsets from liver biopsies across different phases of chronic hepatitis B (CHB), based on data from Zhang *et al.* [2]. The methodological workflow is provided as supplementary material. **a** Identification of major immune cell types using a majority-voting approach with CellTypist v1.7.1 applied to unsupervised clustering. **b** Clustering of B-cell subsets. **c** Annotation of B-cell subsets based on canonical marker gene expression. **d** Expression of unswitched isotype markers. **e** Expression of switched isotype markers. **f** Proportions of B-cell subsets across CHB phases. **g** Summed proportions of *FCRL1* ^+^ B-cell subsets (NBCs, pre-act BCs 1, and pre-act BCs 2) across different groups. No significant differences were observed between the HC and IA groups (unpaired *t* test). **h** Gene set enrichment analysis of *FCRL1⁺* B-cell subsets across different CHB phases. Abbreviations: *Exp* expression, *pre-act BCs* pre-activated B cells*, GC BCs* germinal center B cells, *NBCs* naïve B cells, *MBCs* memory B cells, *HC* healthy control, *IT* immune tolerant, *IA* immune activation, *CR* chronic resolved, *AR* acute resolved

We found that *FCRL1* was predominantly expressed in the naïve B-cell subsets, including non-activated NBCs and pre-activated B cells 1 and 2. Pre-activated B cells show lower expression of *IGHD* than NBCs and lower expression of the activation markers *NR4A1* and *CD83* than activated B cells, and hence, they are reminiscent of NBCs (Fig. 1c). Furthermore, the *FCRL1*^+^ subsets predominantly expressed unswitched isotypes (Fig. 1d + e). Neither were any of the *FCRL1*^+^ subsets individually dominant in IA (Fig. 1f), nor was the summed proportion of *FCRL1*^+^ subsets significantly enriched in the different patient groups, including IA (Fig. 1g). Gene set enrichment analysis on the *FCRL1⁺* B-cell subsets revealed similar enrichment scores for B-cell activation processes in the IA and IT phases (Fig. 1h). The discrepancy with the results by Li *et al.* presumably can be explained by our different bioinformatic approach, *i.e*., we did not apply data integration but dealt with the data set of liver cells separately. Furthermore, we adapted our cluster resolution in a way that the subset classification reflects the observed B-cell heterogeneity in the liver. Like Li *et al.*, we also found enhanced atypical B cells in the IT phase, whereas Osmani *et al.* reported no increase of atypical B cells in liver samples from CHB patients [5]. However, the limited number of B cells detected in the liver makes it difficult to determine whether the observed enrichment patterns reflect biologically relevant signals, or are the consequence of technical limitations, such as the high dropout rate inherent to single-cell RNA sequencing.

In summary, we appreciate the valuable insights into B-cell dynamics across different phases of CHB by Li *et al.* [1]. Our analysis additionally revealed a higher heterogeneity of hepatic B cells in CHB than described in the original publication, and we found that *FCRL1*^+^ B cells not only show signs of activation in the IA, but also in the IT phase of CHB. Additional studies are needed to further investigate the heterogeneity of liver-resident B cells. To conclude about the abundance of *FCRL1*^+^ B cells under conditions of CHB, higher number of cells and larger, representative cohorts need to be investigated.

## Supplementary Information

Below is the link to the electronic supplementary material.

Supplementary file1 (DOCX 29 KB)

## Data Availability

The original data for the analysis provided in this publication was previously published by Zhang *et al.*, 2023 (doi: 10.1136/gutjnl-2021-325915).
