# Supplementary material for: B-cell subset heterogeneity in the liver during chronic hepatitis B
Source: Hepatol Int. 2025 Oct 3;20(2):464–6. doi: 10.1007/s12072-025-10930-3 (PMC13121279; doi:10.1007/s12072-025-10930-3)
Supplement: Supplementary file 1 — Supplementary file1 (DOCX 29 KB) [file 12072_2025_10930_MOESM1_ESM.docx]

**Supplementary file**

**Data acquisition**

Gene expression data were obtained from the NCBI Gene Expression Omnibus (GEO) under accession number GSE182159 (see Table S1). The corresponding V(D)J sequencing data were retrieved from the Genome Sequence Archive (GSA) at [https://ngdc.cncb.ac.cn](https://ngdc.cncb.ac.cn/) under accession number HRA001730.

**Processing of V(D)J sequencing data for integration with gene expression**

Raw FASTQ files from the V(D)J sequencing were processed using Cell Ranger v7.1.0 with the human reference genome (refdata-cellranger-vdj-GRCh38-alts-ensembl-7.1.0). The resulting isotype information for each cell was subsequently integrated with the corresponding gene expression data.

**Clustering and annotation**

The preprocessed gene expression matrix was imported into Seurat v5.3.0, using 2,000 variable features for integration. Batch effects were corrected using Harmony v1.2.3. Clustering was performed using the Leiden algorithm with 50 principal components (PCs) to ensure effective separation of immune cell populations. Cluster annotation was carried out using CellTypist v1.7.1.

Samples containing fewer than 40 B cells were excluded from further analysis. B cells were then subsetted and reclustered using 10 PCs with the Leiden algorithm at a resolution of 0.3 to obtain finer-grained B cell clusters.

**Gene set enrichment analysis**

Gene set enrichment analysis was performed using the ssGSEA method implemented in the escape v2.4.0 R package.

**Software and Environment**

All analyses and visualizations were conducted using R v4.5.0.

**Table S1. Identification of the samples included in the analysis.**

| **ID** | **Donor ID** | **Sample IDs** | **Group** |
| --- | --- | --- | --- |
| HC1 | Dhc570_Liver_1 | GSM5519483 | Healthy control |
| HC2 | D528848_Liver_1 | GSM5519484 | Healthy control |
| HC3 | D529074_Liver_1 | GSM5519485 | Healthy control |
| HC4 | D529351_Liver_1 | GSM5519486 | Healthy control |
| HC5 | D529354_Liver_1 | GSM5519487 | Healthy control |
| HC6 | D529409_Liver_1 | GSM5519488 | Healthy control |
| IT1 | P190604_Liver_1 | GSM5519469 | Immune tolerant |
| IT2 | P190326_Liver_1 | GSM5519471 | Immune tolerant |
| IT3 | P190402_Liver_1 | GSM5519472 | Immune tolerant |
| IT4 | P190910_Liver_1 | GSM5519494 | Immune tolerant |
| IT5 | P190808_Liver_1 | GSM5519495 | Immune tolerant |
| IA1 | P190719_Liver_1 | GSM5519477 | Immune activation |
| IA2 | P190801_Liver_1 | GSM5519496 | Immune activation |
| IA3 | P190911_Liver_1 | GSM5519497 | Immune activation |
| IA4 | P191028_Liver_1 | GSM5519499 | Immune activation |
| IA5 | P191112_Liver_1 | GSM5519502 | Immune activation |
| CR1 | P191126_Liver_1 | GSM5519506 | Chronic resolved |
| CR2 | P191127_Liver_1 | GSM5519508 | Chronic resolved |
| CR3 | P191210_Liver_1 | GSM5519510 | Chronic resolved |
| AR1 | P190716_Liver_1 | GSM5519475 | Acute resolved |
| AR2 | P191008_Liver_1 | GSM5519504 | Acute resolved |
| AR3 | P191217_Liver_1 | GSM5519512 | Acute resolved |
